# An Update on Protective Effectiveness of Immune Responses After Recovery From COVID-19

**DOI:** 10.3389/fimmu.2022.884879

**Published:** 2022-05-20

**Authors:** Saeede Soleimanian, Soheila Alyasin, Najmeh Sepahi, Zahra Ghahramani, Zahra Kanannejad, Ramin Yaghobi, Mohammad Hossein Karimi

**Affiliations:** ^1^ Allergy Research Center, Shiraz University of Medical Sciences, Shiraz, Iran; ^2^ Transplant Research Center, Shiraz University of Medical Sciences, Shiraz, Iran; ^3^ Department of Allergy and Clinical Immunology, Namazi Hospital, Shiraz University of Medical Sciences, Shiraz, Iran

**Keywords:** recovered, hybrid immunity, vaccination, cellular immunity, COVID-19

## Abstract

Severe acute respiratory syndrome coronavirus 2 (SARS-CoV-2) exhibits variable immunity responses among hosts based on symptom severity. Whether immunity in recovered individuals is effective for avoiding reinfection is poorly understood. Determination of immune memory status against SARS-CoV-2 helps identify reinfection risk and vaccine efficacy. Hence, after recovery from COVID-19, evaluation of protective effectiveness and durable immunity of prior disease could be significant. Recent reports described the dynamics of SARS-CoV-2 -specific humoral and cellular responses for more than six months in convalescent SARS-CoV-2 individuals. Given the current evidence, NK cell subpopulations, especially the memory-like NK cell subset, indicate a significant role in determining COVID-19 severity. Still, the information on the long-term NK cell immunity conferred by SARS-CoV-2 infection is scant. The evidence from vaccine clinical trials and observational studies indicates that hybrid natural/vaccine immunity to SARS-CoV-2 seems to be notably potent protection. We suggested the combination of plasma therapy from recovered donors and vaccination could be effective. This focused review aims to update the current information regarding immune correlates of COVID-19 recovery to understand better the probability of reinfection in COVID-19 infected cases that may serve as guides for ongoing vaccine strategy improvement.

## Introduction

About two years after the first identified coronavirus disease 2019 (COVID-19) outbreak, it is still hard to precisely anticipate when the pandemic will finally end, and the protective immunity status in patients after severe acute respiratory syndrome coronavirus 2 (SARS-CoV-2) infection is a global concern (1, 2). Investigating whether cellular immune response and humoral immunity against SARS-CoV-2 are associated with a decreased risk of reinfection could be a vital issue. In light of determining the future dynamics of SARS-CoV-2 circulation, it is critical to clarify how frequently natural infections with SARS-CoV-2 stimulate that level of protection (3). Evaluating longevity to immunity is needed to understand the effectiveness of immunity acquired by natural infection (4).

The underlying mechanisms of lifelong immunity after viral infections have not been unraveled yet. The Smart et al. study showed that antibodies had half-lives of 50 years or more for varicella-zoster and measles; however, antibody levels in response to non-replicating protein antigen, including tetanus and diphtheria, dropped comparatively quickly, suggesting that antigen-specific mechanisms play a crucial role in determining the duration of humoral immunity in an individual (5). Magnitude and persistence are two important factors of humoral response in providing sufficient immune protection (6). Evidence has shown that the humoral response of SARSCoV-2 patients significantly decreased at 1 and 6 months after infection. COVID-19 has a spectrum of clinical complications, from asymptomatic to moderate symptoms and even severe manifestations. Notably, recent studies found that the severe patients had high levels of neutralizing antibodies until 3 months’ post-infection (7).

Some studies proposed that presenting the symptoms of the disease in an individual who has formerly been infected and recovered is unlikely; nevertheless, emerging evidence indicates positive RT-PCR tests post COVID-19 (8–11). Hence, the neutralizing titers were not associated with the viral shedding duration, indicating that humoral immune protection alone might be inadequate and other immune components (T cells and innate immune cells) should also be taken into consideration for achieving virus clearance in SARS-CoV-2 patients (12).

In the previous variants, a few confirmed cases of SARS-CoV-2 reinfection have been reported (13–15). Nevertheless, since the immunity acquired through the previous infection is less effective against Omicron than against other variants, with the Omicron variant surging, the risk of reinfection has become a day-to-day fact (2). The course of reinfection disease has been controversial in the literature regarding the severity of the disease; some show a worse course of the disease (16, 17) and some indicate milder symptoms or no symptoms of reinfection at all (18). It remains unclear whether the severity of the primary disease is related to the risk of reinfection (15, 19), and moreover whether natural immunity after recovery is durable in COVID19 patients with differing severity.

A study demonstrated that asymptomatic or mild symptomatic individuals could not mount virus-specific germinal centers, causing failure in prolonged humoral immunity (1). Alternatively, the mentioned groups of patients mounted effective T helper 1 (Th1) and cytotoxic CD8 + T cells responses. Contrarily, robust induction of virus-specific GC B cell responses and minimally induced virus-specific TH1 and CD8+ T cells were seen in individuals with moderate to severe symptoms (20). Population-level studies show that most individuals who recover develop low levels of neutralizing antibodies (21), which are more significant in preventing reinfection than in the fight against the contracted disease (22).

Some studies revealed that the function and the total number of natural killer (NK) and CD8+T cells were markedly impaired during the early stages of SARS-CoV-2 infection. Evaluation of PBMC in the late stage of recovery patients indicated that the total number of B cells, NK, and T cells went back to normal again. It seems evident that the risk of reinfection must be evaluated in the COVID-19 disease for informing interventions to guide treatment strategies, foresee the disease course, and ascertain whether patients develop long-lasting immunity (23). This review will be helpful to clarify the status of protective immunity in the recovery process of COVID-19 disease and determine the infected cases into separate groups based on the acquired immunity and the possibility of reinfection.

## Humoral Immunity During COVID-19 Infection and Recovery Stage

Humoral immune responses are highly specific and provide long-lasting protection against reinfection (22–25). Antibodies act by either binding to the virus and preventing its interaction with its receptor (neutralizing antibodies) or by causing the destruction of infected cells and the virus bound to them and marking them for demolition through cell-mediated immune response (binding antibodies), which contribute to recognize and initiate the clearance of antibody-coated target cells (26). The ability to recruit antibody-dependent cellular phagocytosis (ADCP), complement-dependent cytotoxicity (CDC), and antibody-dependent cellular cytotoxicity (ADCC) is the activity of the binding antibodies (27). Extra-neutralizing antibody functions are associated with the recovery and prevention of many infectious diseases. Conducting *in vitro* experiments pointed to the infection of macrophages in the absence of ACE2 receptors, which the virus uses to enter the cell (28). This entrance is facilitated by antibody-mediated virus uptake *via* FcγRII6 and is thought to trigger pattern-recognition receptors and induce inflammatory cascades. In addition to FcR expression, almost all innate immune cells present complement receptors providing antibodies with the power to direct the immune system. Antibodies play a critical role in direct anti-viral immunity and priming T cells by delivering antigens to antigen-presenting cells (29).

Antibodies react the same way to SARS-COV-2 as they do to other viruses, with IgM and IgA being the first ones to rise and wane and IgG increasing later on and persisting more prolonged than the previous two. In Wu et al.’s study, multiple antibodies such as immunoglobulin M (IgM) and G (IgG), receptor-binding domain (RBD) of the spike (S) or the nucleocapsid (N) protein, and neutralizing antibodies were evaluated 6 months after the disease onset (30). Specific IgM-S/N are untraceable in the 12th week in most individuals, and IgG-S/Ns titers decrease moderately but reach a plateau at relatively high levels in 6 months with positivity rates for binding and neutralizing over 70%; these findings are in line with those of another study, indicating that IgA and IgM decreased swiftly unlike IgG and neutralizing antibodies which plateaued for 4 months (31, 32). This data fortifies the idea of prolonged humoral immunity after COVID-19 infection (30). Similar studies have indicated that neutralizing antibodies decrease about 3 months after infection (33, 34). In most individuals, the RBD-IgM of S and N proteins was untraceable after 12 weeks. IgG-S/N experienced a decrease and was sustained at high levels in most individuals after 6 months (30, 35), and a protective level prevails over a period of 9 months, up to 1 year (3, 4).

Recovered patients who presented with mild symptoms showed a noticeable rise in the percentage of B cells compared to healthy individuals. Despite specific anti-S IgG in all COVID-19 patients, regardless of how severe the symptoms are, a rise was seen in this antibody level as well (36). It remained high 90 days after the infection in some individuals indicating long-term and steady antibody levels. Intriguingly, the male participants had notably higher anti-S IgG levels after recovery than females, as seen in other works reflecting sex-dependent humoral immune response against SARS CoV-2 (36, 37). There was also a significant positive correlation between the patients’ age and their anti-S IgG antibody levels, showing a greater concentration in older adults compared to younger adults. Patients were seropositive 100 days after the disease onset when the latest measurement occurred (38). Robust humoral immunity correlates with the spike-specific antibodies, memory B cells, and circulating follicular helper T cells (cTFH), steadily induced after SARS-CoV-2 infection and associated with plasma neutralizing activity (39).

The Feng et al. study revealed that receptor-binding domain immunoglobulin (RBD-IgG), full-length Spike-IgG concentrations, and serum neutralizing capacity drop during the first 6 months but remain stable for up to 1 year. Even individuals who had produced high IgG levels during early convalescent stages had IgG levels that had decreased to a similar level 1 year later. Notably, the RBD-IgG level positively correlates with serum neutralizing capacity, suggesting the representative role of RBD-IgG in predicting serum protection (40).

A study investigated the magnitude and significant differences in Ab level which is presented in recovered and naïve individuals. They demonstrated a rise in IgG, IgA, and IgM levels. Among these, elevated S-specific IgG and IgA levels in serum were particularly noticeable. The increase in antibody levels to endemic CoV was more distinct among IgG1 and IgG3 subclasses and was also apparent in nasal and stool samples (41). Given the evidence of robust humoral responses in systemic and mucosal specimens, the neutralization potency of serum and nasal wash specimens was evaluated. Elevated serum neutralization activity was detected in hospitalized subjects who contracted the severe form of the disease. Unlike the serum samples, nasal specimens from subjects with the severe disease showed little to no viral neutralization. Individuals with increased mucosal neutralization activity reported milder symptoms more frequently than those who presented with more severe forms of the disease. Interestingly, robust nasal and serum neutralization activities were not co-induced (41).

Isho et al. reported that anti-SARS-CoV-2 IgA and IgM antibodies decreased quickly, whereas IgG antibodies were steady for up to 105 days’ post symptom onset in serum and saliva. They indicated that IgG, IgM, and to a lesser extent, IgA levels in the serum are directly correlated with matched saliva specimens (31).

**Figure 1:**
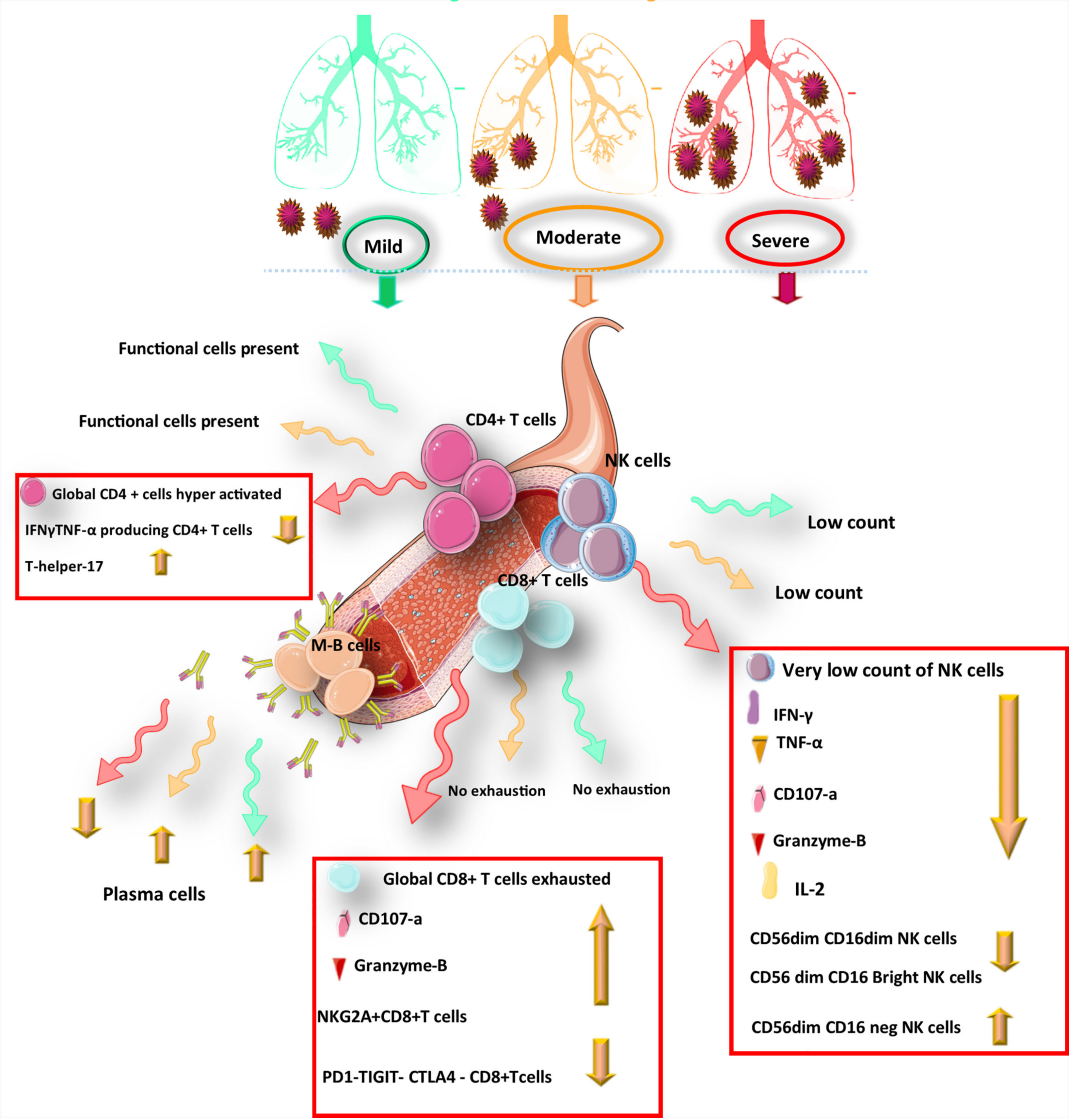
Schematic representation of the protective immunity against COVID-19 infection during infection.

Although many studies have mentioned the levels of different antibodies participating in the COVID-19 course, one should consider the required titers for sustained immunity, which is not yet determined for COVID-19. Higher titers do not necessarily mean more protection against reinfection and may be because of higher antigen exposure (29) or an indicator of the severity of the disease (35). However, some studies suggest that the quantity of antibodies that persist is directly related to the extent of protection against the virus that induced them (42). Another key contributor to consider in prolonged immunity is memory lymphocytes. In the convalescent period of a viral disease, when the antigen is no longer present, and antibodies diminish, memory B cells wait around in the bone marrow for reinfection. The memory B cells then differentiate into plasma cells and produce antibodies (**Figure 1**). Several extensive studies have indicated a marked increase in plasmablast count in peripheral blood of COVID-19 patients (43–46). The data on memory B cells in COVID are still lacking. However, persisting memory B cells in individuals with mild symptomatic recovered COVID- 19 infection (34) suggest possible longevity of the mentioned cells (35).

Studies propose two main paths toward long-term humoral immunity against diseases; memory B-cell–dependent antibody production by short-lived plasma cells and memory B-cell–independent antibody production by long-lived plasma cells (47–53). Theories of memory B-cell dependence need a correlation between memory B cells after each infection and antibodies, which has been controversial (54–57). Studies indicated that memory B cells and antibody production are regulated independently, paving the long-lived plasma cell path (5, 54, 58, 59), and extended survival and antibody secretion time to over 1 year in COVID-19 (5), and SARS-CoV-2-specific B cell immunity persists despite overall antibody decline (60). Indeed, production of memory B-cells after every immunization dose or natural immunity did not correlate necessarily with antigen-specific antibody levels. Memory B-cells remain elevated over several years; while there is a rapid decline in neutralizing antibodies, although the disappeared antibodies did not mean without immune protection, given that memory B cells might rapidly initiate a new immune response when the virus is reencountered.

## CD4^+^ and CD8^+^ T Cells During COVID-19 Infection and Recovery Stage

Lymphocytopenia has been observed in patients with different levels of COVID-19 severity (61–65). According to studies, the severity of the COVID-19 disease is positively correlated with the increased level of inflammatory cytokines while inversely correlated with lymphocyte numbers, especially in patients with severe and critical symptoms (61, 64–68). T cells exhaustion occurs during disease progression (66) (63, 65, 69). Notably, in severe disease patients, all lymphocyte subsets were reduced, whereas in mild or asymptomatic individuals the accounts of NK, NKT, and γδ -T cells were similar to healthy individuals or even higher than them (70).

During COVID-19 infection, especially in the stage of disease progression, increased expression of inhibitory immune checkpoints, including programmed death (PD-1), PD-L1, T-lymphocyte associated protein-4 (CTLA-4), T cell immunoglobin, and mucin protein 3 (TIM-3) on T cell surface renders T cell exhaustion and dysfunction, disabling T cell-mediated anti-viral immunity (66, 71). Emerging evidence indicates that co-blockade of TIM3 and PD1 can recover the effector function of T cells (72, 73). Indeed, TIM3 acts as a checkpoint receptor expressed on ‘exhausted’ T cells and that inhibition of TIM3 boosts the effect of PD1 blockade (74).

The current study of hospitalized patients with COVID-19 infection indicated that higher expression of PD-1 and Tim-3 were observed in CD8+ T cells in COVID-19 patients requiring ICU care. Similarly, Tim-3 expression enhanced in CD4+ T cells during severe and ICU period disease stages while PD-1 expression in CD4+ T cells was not overtly changed toward the disease progression (66). Hence, co-blockade of TIM3 alongside inhibition of other checkpoint receptors such as PD-1 could be therapeutic potential targets to treat SARS-CoV-2 infection. Furthermore, NKG2A is an inhibitory receptor that expresses on NK cells and CTLs, blocking anti-virus activity of cytotoxic lymphocytes. In recovery patients, restoration of immune cells count occurs with downregulation of NKG2A expression, which suggests that the progression of COVID-19 disease with cytotoxic lymphocytes exhaustion may result from upregulation of NKG2A in the early stage of COVID-19. During COVID-19 infection, over-expression of NKG2A also leads to decreased production of CD107a, IFN-γ, IL-2, granzyme B, and TNF-α (**Figure 1**), which are necessary for cytotoxicity function (75).

**Figure 2:**
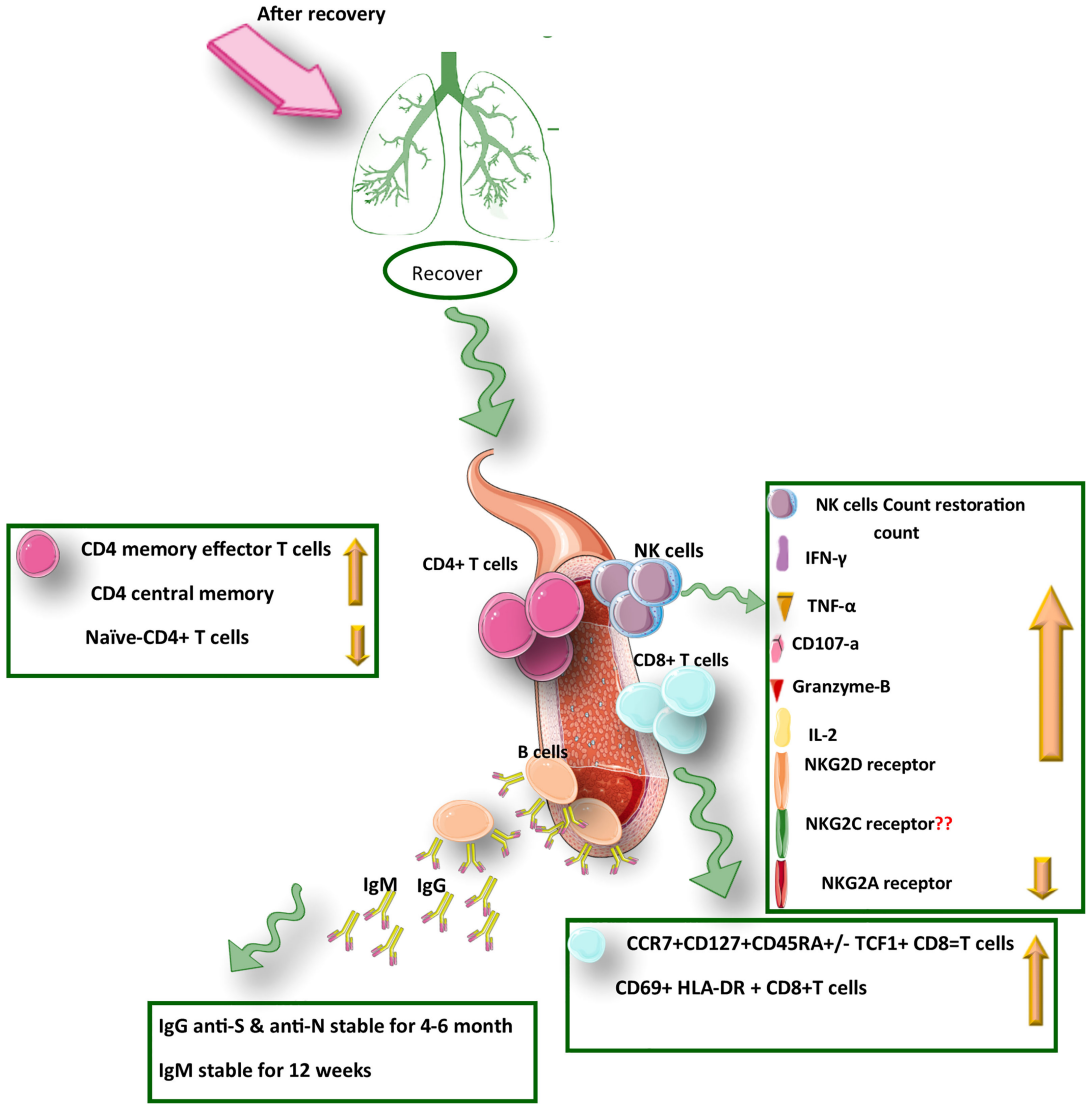
A summary of the protective immunity against COVID-19 infection after recovery from SARS-CoV-2 infection.

The whole transcriptome evaluation of innate, humoral, and cellular immunity in mild, moderate, and severe COVID-19 patients during three different time points (treatment, convalescence, and rehabilitation) showed that after recovery in an infected individual, the strong protective response by T cells through generating memory T-cells pool against SARS-CoV-2 has been induced regardless of the severity of the disease. In contrast, poor innate and humoral immune responses have been observed. In addition, in recovered COVID-19 patients, activation of transcription and differentiation genes of T cells was detected, approving the strengthening of the T cells’ immune response at this stage, which indicated an increased level of CD4+ memory/effector and CD4+central memory T cells after COVID-19 recovery. It was revealed that the level of CD4+ memory T-cell in the recovery phase was associated with the severity of the disease (63). Memory CD8+ and CD4+ T cells have a similar cluster of markers of activation/cycling phenotype, including CD38, Ki-67, HLA-DR, and PD-1; however, the results of the flow-cytometry analysis showed an increased number of CD4+ T cells after recovery, while CD8+ numbers remained unchanged (76). It was found that CD8+ T cells in the infectious stage of the disease with limited proliferation had the Ki-67, CCR7- CD27+ CD28+ CD45RA- CD127- phenotype, which was in the convalescent-phase; these cells tend to differentiate toward memory cells (withCCR7+ CD127+ CD45RA-/+ TCF1+ phenotype) (**Figure 2**). This revealed differences of the memory cells between the infection and recovery stage (62).

To evaluate the functional capabilities of memory CD8+ and CD4+ T cells in convalescent COVID-19, Sekine et al. used nucleocapsid, Spike, and membrane peptides to stimulate PBMCs. They showed that SARS-CoV-2-specific CD4+ T cells express IFN-γ, IL-2, and TNF-α, while CD8+ T cells are characterized by IFN-γ production and mobilized CD107a expression. Notably, Spike-specific-CD4+ T cells were skewed toward circulating T follicular helper, while membrane and nucleocapsid specific CD4+ T cell were differentiated toward Th1 or a Th1/Th17 cells (62, 77, 78). Moreover, a study revealed that in hospitalized patients there was increased levels of specific IgG (against Spike and RBD), and memory B cells; while CD4+ memory T cell numbers were decreased compared with non-hospitalized ones (79). These results indicated a significant role of cellular immunity in severe COVID-19 disease. Furthermore, after recovery, circulating CD8+ and CD4+ memory T cell numbers were monitored for 1 month and 6 months after COVID-19 infection, showing a trend toward decreasing both subsets after 6 months (79). SARS-CoV-2-specific CD4+ T cells were Th1 cells and predominantly T central memory (Tcm) cells with robust helper function phenotypic features. A kind of CD4+ memory cell subset observed in COVID-19 infection is T follicular helper (cTFH) memory cells with shelf life of more than 6 months after disease onset (79).

Most SARS-Cov-2-specific CD8+ T memory cells differentiated to effector memory cells re-expressing CD45RA (Temra) T-cells with less terminal differentiation than most Temra cells (80). Overall, the cooperation of all parts of the immune system is essential to fight infection and protect the body against re-infection. Interaction between humoral and cellular memory cells leads to long-term solid adaptive protection (79, 81). Cross-reactive memory T cells of human coronaviruses have challenged the evaluation of COVID-19 specific memory cells responses (81). As the immune responses to infection depend on the patient’s immune system and vary from patient to patient, likewise, the immunity in recovered patients and the functions of their memory cells will vary from person to person. Having considered that regulating cytokine productions is important in the inflammation and functions of immune cell types, cytokine profiles of patients have been studied in the course of infection and during recovery.

It was reported that IL-2, IL-7,IL-6, IL-10, TNF-α, TGF-β, G-CSF, IP-10, MCP-1, and MIP-1A rose in COVID-19 patients, especially in ICU patients compared to non-ICU patients (82, 83). Based on Diao et al.’s report, the origin of these cytokines is not T cells and revealed that some cytokines are released from monocytes and macrophages. Secretion of cytokines effects on immune responses. TNF-α leads to reduced T cells, or IL-6 causes antibody production and effector T-cell development, but IL-10 results in T cell exhaustion and prevents T cell proliferation. This finding refers to the inverse effects of serum cytokines level on the survival and proliferation of T cells. Remarkably, normal cytokine levels in some ICU patients may indicate immunodeficiency (66). Evidence demonstrated that after recovery some cytokines such as IL-10, TNF-α, and IL-6 were significantly decreased in the late recovery stage compared to the early recovery stage, while TGF-β level was not significantly different between the early and late stages of recovery. However, the TGF-β reduction was observed during a recovery (84).

Generally, evaluation of recovered COVID-19 patients showed that months after infection, a strong response of memory T cells against the SARS-Cov-2 spike, membrane, and nucleocapsid peptides could be detected even in the absence of circulating antibodies (62). Nevertheless, re-infection with SARS-CoV-2 occurs (85, 86). It is also possible that COVID-19 recovered patients with lower memory cells will be more susceptible to re-infection (79).

## NK Cells During COVID-19 Infection and Recovery Stage

NK cells are early effector cells that play an essential role during viral infections. Several studies have indicated that during COVID-19 infection from moderate to severe infection, peripheral NK cells were dropped (87). The frequency of NK cells decreased remarkably in severe patients compared with those in mild cases and healthy controls (88). The key question arises whether impairment of NK response, followed by increased susceptibility to reinfection, is a concern for recovered patients. Effective response against viral infection needs the cooperation between humoral and cellular immunity. Yunbao Pan et al. found that in COVID-19 convalescent individuals, the production of NAbs might be correlated with the NK cells’ antiviral activity as well as the activation of T cells (89).However, NK cells are critical pieces of this puzzle; the relative significance of these cells remains unclear.

According to a study, the frequency of CD56+ CD16+ NK cells in the asymptomatic patients was significantly different from that in healthy individuals after recovery, displaying an expansion the level of NK cells in the asymptomatic COVID-19 patients. Hence, an important role could be assigned to NK cell immunity which is sustained after recovery from COVID-19 (70). Notably, as an inhibitory receptor, the NKG2A receptor on NK cells has been indicated to cause NK cell exhaustion in chronic viral infections (90). In the early stage of COVID-19, disease progression is associated with the functionally exhausted NK cells in patients infected with SARS-CoV-2. Recently it was reported that in COVID-19 patients, the expression of NKG2A was elevated significantly on cytotoxic lymphocytes compared with that in healthy individuals (75). Interestingly, the NKG2A expression on both NK and CD8+ T cells was reduced after recovery (**Figure 1**). Moreover, in patients convalescing after viral therapy, the percentages of both NKG2A+ CTLs and sequentially NKG2A NK cells were also dropped, indicating decreased expression of NKG2A could be an indicator for the useful control of SARS-CoV-2 infection (91). Therefore, efficacious treatment is accompanied by fewer NKG2A+ NK cells and TCD8+ cells along with the restoration of lymphocytes percentage, including NK cells. Regarding treatment through inhibition of roadblocks to immune tolerance could be important in the formation of COVID-19 persistence. After identification of NK cells’ functionality through the assessment of CD107a and granzyme B(cytotoxicity markers) alongside the evaluation of IFN-γ, TNF-α, and IL-2 levels (inflammatory proteins), it was revealed that upon COVID-19 infection, the exhausted status of cytotoxic cells was also reflected in decreased production of cytotoxic effector molecules, including CD107a, IFN-γ, IL-2, Granzyme B, and TNF-α, which was resorted and gone up after therapy in convalescent individuals (92).

Based on the surface expression of CD56 and CD16 receptors, NK cells are subdivided to different subsets with distinct functions; the first subset, CD56dimCD16pos cell, includes cytotoxic cell, the second subset is CD56brightCD16neg, which is considered as a producer of cytokines, and the third subset is CD56dimCD16neg, the unconventional subset, which expands in different pathological conditions (93). On the one hand, it has been revealed that NK cells in patients with severe COVID-19 are deficient and impaired. On the other hand, the enhanced presence of NK cells in bronchoalveolar lavage (BAL) has been found. One reason for decreased NK cells could be the homing of NK cells from peripheral blood to the lung. Besides, the CD56dimCD16pos NK cell subset with increased KIR expression was found in the lungs (94). Notably, immune-modulating occurs through KIR receptors of NK cells, playing a significant role against SARS-CoV-2 infection (95), and expression of CC chemokine receptor **(**CXCR) 3, CXCR6, CCR5 on NK cells could cause the homing event developing in BAL of COVID-19 patients. In this regard, these markers, especially CCR6, could be considered risk factors in developing severe COVID-19 (72).

Despite NK cell depletion, the evaluation of different distributions of NK cell subsets could be appreciated in COVID-19 patients compared to convalescent individuals since these changes in the distribution of NK cells phenotype could be assigned as a cause of NK cell defense role impairment upon COVID-19 infection (93, 94). It might give us a tool to improve the diagnostic evaluation and the immunized and non-immunized COVID-19 patients’ estimation. Yet, further studies are required to clarify the role of NK cells in individuals with COVID-19.

Moreover, the assessment of NK cells based on CD56, CD16, and KIRs expression in hospitalized COVID-19 patients indicated that a higher frequency of KIR2DL1 inhibitory receptor was concomitant with reduction of CD56dimCD16dim and CD56dimCD16bright NK Cell Subsets. Interestingly, this outcome was in parallel with CD56dimCD16neg NK Cell Subset expansion and higher frequency of KIR2DL1 and KIR2DL1/S1 inhibitor receptors (95). In line with this result, there is NK cell’s ADCC activity that results from the expression of CD16 on NK cells as an FC receptor, allowing the detection of antibody-coated infected and cancerous cells (72), hence the lack of CD16 expression could be associated with COVID19 severity. Recently several features of immunological memory have been found in NK cells similar to B and T cells, such as long-lived progeny expansion, education clonal-like generation, and robust secondary responses (74). It was revealed that NK cells have displayed their distinctive cytotoxic ability against viral infections, including CMV, hantavirus (74), chikungunya virus (74), and cancerous cells, along with the upregulation of NKG2C activating receptor, raising the possibility of a distinct subset of NK cell which could selectively respond against a certain target and then driving memory (60). In this line, it was mentioned in our previous study that introducing NKG2C into chimeric antigen receptors in NK cells to enhance effector functionality might be a potential approach in future viral immunotherapy for emerging and re-emerging viruses (42). Indeed, investigating this memory potential of NK cells against specific pathogens might be efficient for targeted cell therapy and vaccine development. There are several clinical trials using NK cells for cell therapy as an off-the-shelf living drug in the treatment of COVID-19 infection (75).

### Immunity to SARS-CoV-2 Variants of Concern

Both natural infection with SARS-CoV-2 and immunization with vaccines elicit protective immunity. However, the extent to which such immune responses protect against emerging variants is of increasing importance. Such VOC include Alpha (B.1.1.7), Beta (B.1.351), Gamma (P.1), Delta (B.1.617.2), and a new one, Omicron (B. 1. 1. 529).

In late 2021, the Omicron virus variant emerged, with significant genetic differences and clinical effects from other variants of concern. This variant demonstrated higher numbers of polymorphisms in the gene encoding the Spike (S) protein, and there has been displacement of the dominant Delta variant (96). Natural infection with SARSCoV-2 induces robust protection against re-infection with the B.1.1.7 (alpha),1,2 B.1.351 (beta),1 and B.1.617.2 (delta)3 variants. However, the B.1.1.529 (omicron) variant makes multiple mutations that can provide immune evasion (97). Indeed, SARS-CoV-2 Omicron variant pseudovirus exhibits escape from vaccine-induced humoral immunity. In addition, pseudovirus produced with the Omicron spike exhibited more efficient transduction of ACE-2 expressing target cells than other variants. A study reported a near-complete lack of neutralizing activity against Omicron in polyclonal sera from individuals vaccinated with two doses of vaccine and from convalescent individuals, as well as resistance to different monoclonal antibodies in clinical use (98), highlighting the global need for vaccine boosters to combat the impact of Omicron and emerging variants (99, 100). However, Omicron is strongly neutralized by antibodies induced by booster third dose vaccination (101, 102) or fourth dose in hemodialysis patients (103) or by heterologous vaccination.This has led to increased attention to the important role of T cells in protection immunity. It was revealed that the responses of Spike-specific CD4^+^ T cells were not different in variants of concern; while CD8^+^ T cells responses to Omicron spike were reduced compared to other variants, enhanced with booster vaccine doses (104).

A national database study in Qatar found that there is a strong protection against re-infection with the Alpha, Beta, and Delta variants of SARS-CoV-2 (at approximately 90%), which is confirmed with previous studies. While such effectiveness in preventing reinfection with the Omicron variant was lower (approximately 60%), however, it was still significant. Furthermore, regardless of variant, the immunity of previous infection against hospitalization or death caused by reinfection appeared to be effectiveness (105).

## Protection of Natural Immunity, Vaccine Immunity, Or Hybrid Immunity–Which One Is Better?

It is important to determine the duration and quality of the adaptive immune system, which may be different between natural immunity (obtained by COVID-19 infection) and immunity resulting from vaccination (106). There are several conflicting reports about the immunity of both paths. Evidence indicates infection-acquired immunity reduces with time since the previous infection but prompts longer-lasting immunity against re-infection than mRNA vaccine (7, 107, 108). Conversely, in recovered patients with COVID-19, the risk of SARS-CoV-2 re-infection and even hospitalization persisted low for several months; nevertheless, vaccination affords further protection by a slight difference (12, 109). However, both SARS-CoV-2 infection-derived immunity and vaccination prompt multifaceted, functional immune memory; some studies have highlighted that vaccination–derived immune response following natural immunity boosts the immunity, termed hybrid immunity (6, 110). A study carried out by Goldenberg et al. (84) demonstrated that infection by the Delta variant of SARS-CoV-2 has led to a more potent immune response as compared with the BNT162b2 two-dose vaccine-derived immunity, meanwhile; patients who were recovered from SARS-CoV-2, and were then vaccinated by a single dose of the vaccine acquired increased protective response against the Delta variant. There seem to be specific memory lymphocytes, both B cell and T cell components, to hybrid immunity. It is indicated that in the context of reinfection after natural immunity alone or vaccination of naïve individuals, there is a reduction in the level of antibody-mediated immunity against variants of concern (VOCs) (111), but after one dose of vaccination following the previous infection with former VOCs the immunity rises. It should be noted that neutralizing antibody drops are not due to low antigenicity and spike protein mutations of the VOCs. It is exemplified in a study that found in re-infected patients with B.1.351(Beta) variant (previously infected with non-B.1.351), neutralizing antibodies against this variant after vaccination were shown to be 25 times higher than after vaccination (no involved B.1.351 spike) (112, 113). When natural immunity to SARS-CoV-2 is combined with vaccine-induced immunity, it has been found that higher SARS-CoV-2 RBD-specific memory B cells and variant-neutralizing antibodies and a specific population memory SARS-CoV-2 spike-specific CD4+ T cells than previously naive individuals are generated (114). In this line, the production of diverse memory B cells needs T cells and their cytokine profile. Even if the function of antibodies neutralizing is failed against variants, memory T cells can recognize SARS-CoV-2 variants (99), and in hybrid immunity, T cell memory consists of both spike and non-spike T cell memory, unlike the vaccine-induced memory T cell which involves spike –memory T cells. Furthermore, the mutation does not occur in most epitopes of T cells in new variants, demonstrating that the protective role of T cells’ immunity is preserved (115).

## Conclusion

During recovery, the investigation of cellular and humoral immunity among COVID-19 patients with different disease manifestation could run additional insights into the roles of these cell types during natural host immunity. In addition, the clarification of the recovery and immunity process leads to making the proper decisions by policymakers for screening and lockdown, and improved diagnostic assessments of re-infection in individuals. A combined natural/vaccine immune response to SARS-CoV-2 seems to be a notably potent accompanist. According to this concept, more investigation of combinations of SARS-CoV-2 vaccines with different platforms, such as mRNA and adenoviral vectors or mRNA and recombinant protein vaccines, could be appreciated. Moreover, the breadth of recognition of epitopes through T cells, both CD8 and CD4 lymphocytes, may guide ongoing vaccine strategy improvement. In addition, the study of NK cells alongside the evaluation of cytokine profiles (116, 117) in the hybrid immunity can offer information for understanding which vaccines can cross that threshold of hybrid status to confer individual and herd immunity. Since hybrid immunity may be a reproducible way to enhance immunity, the combination of plasma therapy from recovered donors and vaccination could be effective; however, it needs further support from future studies for selecting the best donors to produce off-the-shelf living drugs.

## Author Contributions

SS devised the main conceptual ideas and designed the figures. SS, NS, and ZGH wrote the manuscript with input from all authors. SS devised the main conceptual ideas and designed the figures. SS, NS, and ZGH wrote the manuscript with input from all authors.

## Funding

This study was supported by grants from Shiraz University of Medical Science, Shiraz, Iran (Grant number: 19640).

## Conflict of Interest

The authors declare that the research was conducted in the absence of any commercial or financial relationships that could be construed as a potential conflict of interest.

## Publisher’s Note

All claims expressed in this article are solely those of the authors and do not necessarily represent those of their affiliated organizations, or those of the publisher, the editors and the reviewers. Any product that may be evaluated in this article, or claim that may be made by its manufacturer, is not guaranteed or endorsed by the publisher.
